# Adenoidal enlargements and associated disease in children

**DOI:** 10.1186/2045-7022-5-S4-P35

**Published:** 2015-06-26

**Authors:** Felicia Manole

**Affiliations:** 1 Faculty of Medicine Oradea, Oradea, Romania

## Introduction

Adenoid enlargement in the pediatric population is a common disease in the pediatric ENT field. Nasopharyngeal airway obstruction by adenoidal tissue was studied in children with otitis media with effusion, chronic suppurative otitis media, rhinosinusitis, snoring, chronic purulent rhinorrhea and oral breathing and chronic cough.

## Material and method

The study included 126 children aged between 3 years and 16 years in 2012-2014 in the Municipal Hospital of Oradea, with adenoidal enlargement who presented with the following associated diseases: 46 with bilateral serous otitis media, 10 with chronic suppurated otitis media, 24 with chronic rhinosinusitis, 32 with allergic rhinitis, 14 with the predominant symptoms chronic cough all of whom were scheduled for adenoidectomy. We preoperatively evaluated the following: nasal obstruction, mouth breathing, close rhinolalia, degree of hearing loss, level of IgE, and type of cough. We preoperatively performed fiber optic examination of the adenoidal tissue in nasopharynx in selected cases.

## Results

All the patients in whom we performed adenoidectomy were evaluated 6 months later to appreciate the evolution of the associated disease. In 64% of patients with serous otitis media the aspects of tympanic membrane were normal, cough disappeared in 76% of the patients, and acute episode of rhinosinusitis were without recurrence. The patients with allergic rhinitis after adenoidectomy suffered from repeated or persistent episodes of hearing loss according to nasal obstruction and congestion from allergens.

## Conclusion

Adenoidectomy in children is performed after general criteria, the history of the medical disease being very important, the incidence of acute episodes and recurrence of different types of infection in ENT field, without a possibility to establish the most important symptom to be predictive for surgery, because all the patients are individual. In the age group between 3 and 6 years, serous otitis media is predominant as is oral breathing. Rhinosinusitis appear to be more predominant in the age group between 6 and 10 years. We cannot establish a relationship between the dimension of adenoidal tissue and associated disease, but, associated diseases like allergic rhinitis have an important prognostic role.

